# Artificial Intelligence Application in Skull Bone Fracture with Segmentation Approach

**DOI:** 10.1007/s10278-024-01156-0

**Published:** 2024-07-01

**Authors:** Chia-Yin Lu, Yu-Hsin Wang, Hsiu-Ling Chen, Yu-Xin Goh, I-Min Chiu, Ya-Yuan Hou, Kuei-Hong Kuo, Wei-Che Lin

**Affiliations:** 1 Department of Diagnostic Radiology, Chang Gung Memorial Hospital, Kaohsiung, Taiwan; 2 Division of Medical Image, Far Eastern Memorial Hospital, No. 21, Sec. 2, Nan Ya South Road., Banqiao District New Taipei City, Taiwan; 3 Department of Neurology, Shuang Ho Hospital, Ministry of Health and Welfare, Taipei Medical University, New Taipei City, Taiwan; 4 Department of Emergency Medicine, Chang Gung Memorial Hospital, Kaohsiung, Taiwan; 5 Department of Neurology, Kaohsiung Chang Gung Memorial Hospital, Kaohsiung, Taiwan; 6 Department of Radiology, Jen Ai Chang Gung Health Dali Branch, Taichung, Taiwan; 7 School of Medicine, National Yang Ming Chiao Tung University, Taipei, Taiwan

**Keywords:** Artificial intelligence, Skull bone fracture, AI vs human comparison, Retrospective studies, Segmentation, Classification

## Abstract

This study aims to evaluate an AI model designed to automatically classify skull fractures and visualize segmentation on emergent CT scans. The model’s goal is to boost diagnostic accuracy, alleviate radiologists’ workload, and hasten diagnosis, thereby enhancing patient outcomes. Unique to this research, both pediatric and post-operative patients were not excluded, and diagnostic durations were analyzed. Our testing dataset for the observer studies involved 671 patients, with a mean age of 58.88 years and fairly balanced gender representation. Model 1 of our AI algorithm, trained with 1499 fracture-positive cases, showed a sensitivity of 0.94 and specificity of 0.87, with a DICE score of 0.65. Implementing post-processing rules (specifically Rule B) improved the model’s performance, resulting in a sensitivity of 0.94, specificity of 0.99, and a DICE score of 0.63. AI-assisted diagnosis resulted in significantly enhanced performance for all participants, with sensitivity almost doubling for junior radiology residents and other specialists. Additionally, diagnostic durations were significantly reduced (*p* < 0.01) with AI assistance across all participant categories. Our skull fracture detection model, employing a segmentation approach, demonstrated high performance, enhancing diagnostic accuracy and efficiency for radiologists and clinical physicians. This underlines the potential of AI integration in medical imaging analysis to improve patient care.

**Supplementary Information:**

The online version contains supplementary material available at 10.1007/s10278-024-01156-0.

## Introduction

Emergency departments manage a substantial volume of traumatic brain injury cases, often presenting with concurrent intracranial hemorrhages and skull fractures [1]. Despite considerable research dedicated to applying deep learning to intracranial hemorrhages [2], studies into AI (artificial intelligence) applications for diagnosing skull fractures remain relatively limited [3, 4]. Skull fractures may lead to critical complications such as intracranial and orbital injuries, cerebrospinal fluid leakage, cranial nerve palsies, and vascular injuries [5, 6]. However, the inherent complexity of multiple fractures complicates the image interpretation [7–10]. AI models have shown remarkable accuracy and augmented diagnostic precision across various medical fields [11, 12].

Earlier research indicates that segmentation for skull fracture detection exhibits superior sensitivity (recall) and specificity compared to object detection.[5, 13] However, a significant portion of AI research related to skull fractures has excluded pediatric and post-craniectomy patient [11, 14–17]. To our knowledge, no preceding studies have conducted a comparative analysis of the diagnostic duration within the scope of skull fractures [18].

In this study, we aim to evaluate an AI model designed to conduct binary classification of skull fractures as well as provides a graphic segmentation mask that clearly outlines the fracture lines. Pediatric and post-operative individuals were not excluded from our training dataset. Moreover, we have undertaken an analysis of the diagnostic duration to further understand its implications.

## Materials and Methods

### Study Design and Data Curation

We retrospectively reviewed brain CT scans (computed tomography) gathered from Far Eastern Memorial Hospital over a 12-year period (January 1, 2010, to January 14, 2022). We assembled 131,410 brain CT reports, which were randomized and checked to ensure the exclusion of any duplicates. Among these, there were 5281 cases confirmed positive for fractures, while 126,129 cases showed no evidence of fractures. Any case that was reported without the keyword “fracture” was considered a fracture-negative instance.

**Fig. 1:**
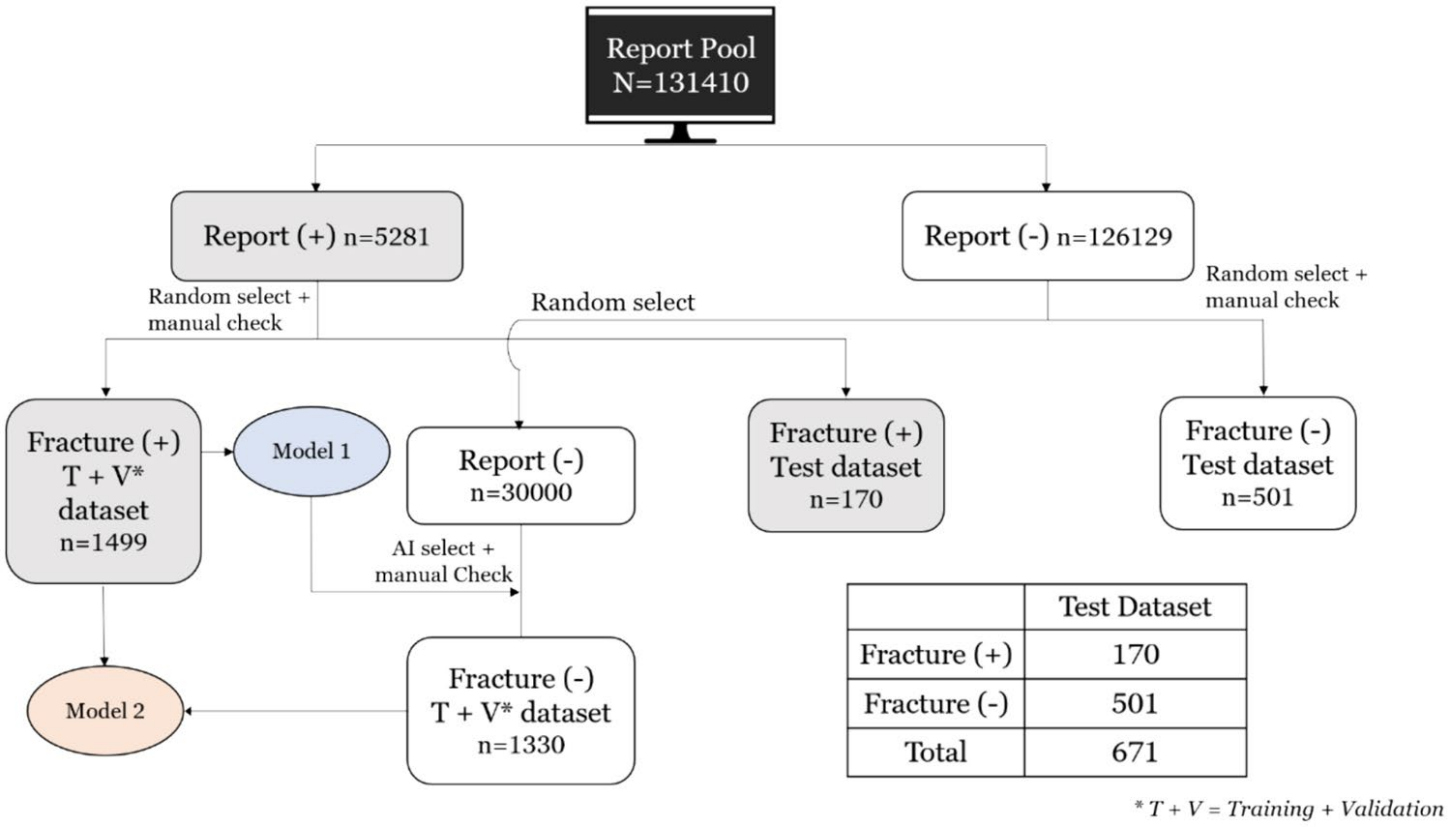
Data selection workflow. This flowchart shows the resources of the training and validation datasets. Model 1 was trained using only fracture positive cases, while model 2 was trained using additional false-positive cases from Model 1. The bottom right corner shows the number of fracture-positive and fracture-negative cases in the test dataset

To evaluate the AI model’s performance independently, we retrospectively collected a separate test dataset randomly comprising 671 cases, including 170 fracture-positive cases and 501 fracture-negative cases. The data selection workflow is shown in Fig. 1.

We selected the first 1499 fracture-positive cases for training Model 1. The 1499 fracture-positive cases were randomly distributed into a training dataset and a validation dataset following a ratio of 12:5. These cases were employed for Model 1 training.

Following Model 1’s training phase, 30,000 fracture-negative cases were randomly selected for Model 1 selection. Manual checks were also performed to ensure that there were no false negatives in the ground truth. Out of the 30,000 negative cases, Model 1 incorrectly identified 1330 cases as false positives. These false-positive cases, along with the initial 1499 cases, were utilized for Model 2 training. Comparative case-wise and slice-wise presentation of training and validation datasets is shown in Table 1.

**Table 1:** Comparative case-wise and slice-wise presentation of training and validation datasets

		Case-wise			Slice-wise		
		Definition	Training dataset	Validation dataset	Definition	Training dataset	Validation dataset
Model 1	Fracture ( +)	Positive study	1166	333	Positive slice	16,403	6998
	Fracture ( −)	Negative study	0	0	Negative slice	16,529	7052
Model 2	Fracture ( +)	Positive study	1166	333	Positive slice	16,403	6998
	Fracture ( −)	False-positive studies from Model 1	1034	296	False-positive slices from Model 1	27,275	11,445

This table shows the total slice number from both positive and negative cases. Model 1 was trained using all positive cases, including the negative slices. On the other hand, Model 2 was trained using an additional 1330 false-positive cases from Model 1, resulting in a total of 27,275 negative slices and 11,445 negative slices

### CT Acquisition and Preprocessing

**Fig. 2:**
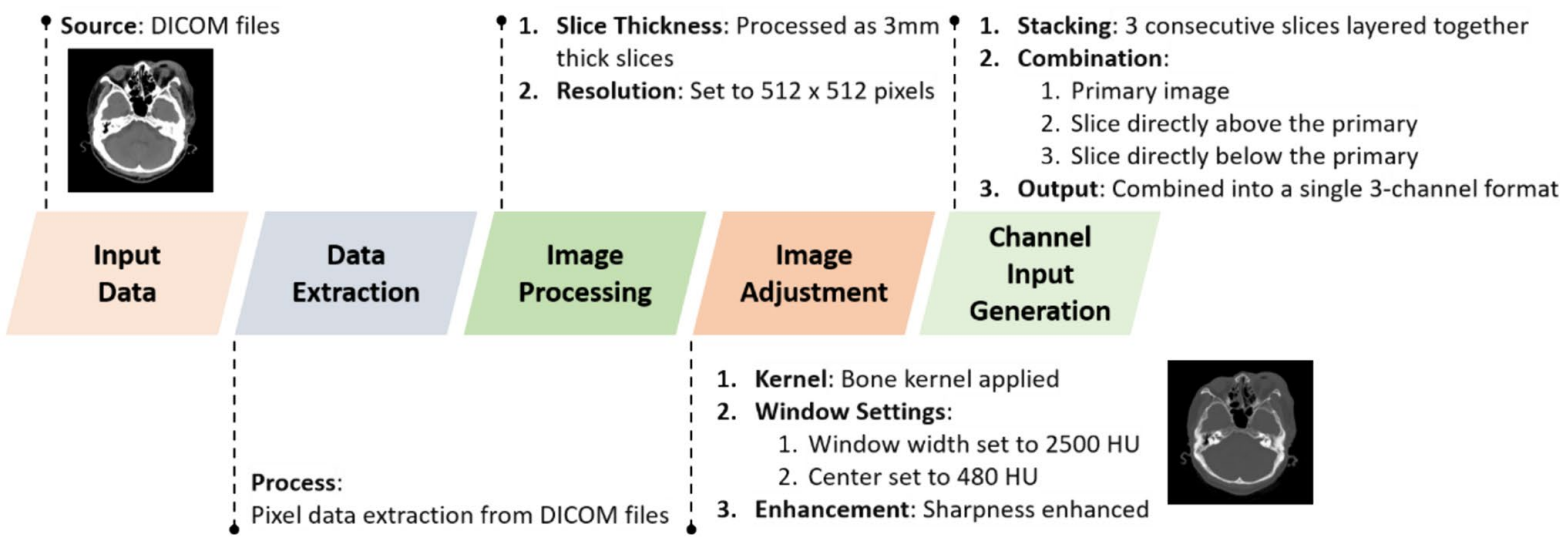
Flowchart of CT acquisition and preprocessing. The input data comprised DICOM files from which the pixel data was extracted. Images were processed as 3-mm-thick slices with a resolution of 512 × 512 pixels and were adjusted using a bone kernel with the bone window settings (window width of 2500 HU and center of 480 HU) enhanced for sharpness. To generate a three-channel input, three consecutive slices were layered together. Essentially, the primary image, along with the slices directly above and below it, was combined into a single three-channel format

The input data files were DICOM files, from which the image data was extracted as pixel arrays. The images were inputted as 3-mm slices with a matrix size of 512 × 512 pixels and transformed to bone kernel with bone window (window width and center are 2500 HU and 480 HU) and sharpening. To create a three-channel input data, three adjacent slices were stacked. In other words, the original image itself, along with the two images above and below it, is placed into a three-channel container. The flowchart of CT acquisition and preprocessing is shown in Fig. 2.

### Reference Standard and Manual Annotation of Skull Fractures

For ground truth, reference standards were reviewed by three radiologists with up to 17 years of experience on a per-case basis. If there is a divergence of opinion among the three radiologists, the majority view will be considered the conclusive diagnosis. Per-slice labeling was done using an image annotation software tool called “ITK_SNAP” version 3.8.0 by a neuroradiologist up to 13 years of experience [19]. The correct detection for each case was determined based on the DICE (Dice coefficient) score, with a threshold of more than 0.15.

The decision to set the threshold at 0.15 for our model’s predictions was the outcome of a meticulous review process undertaken by a group of physicians. These experts conducted a detailed evaluation of the model’s performance across a range of intervals—ranging from 0.9–1.0 down to lower intervals in decrements of 0.1. It was determined that predictions with an interval exceeding 0.15 provided actionable insights, effectively informing the classification and likely location of the identified anomalies. Conversely, predictions falling below this threshold were found to be inconsistent and lacked the necessary specificity for reliable clinical application. After extensive discussions that leveraged the collective expertise and clinical experiences of the team, a consensus was reached. It was agreed that a threshold of 0.15 struck the optimal balance between sensitivity and specificity, thereby maximizing the model’s practical utility in a clinical setting (Table 2).

**Table 2:** Criteria for binary classification using segmentation approach

AI mask	GT mask	DICE	Results
( +)	( +)	DICE ≥ 0.15 1	TP
( +)	( +)	DICE < 0.15	FN
( −)	( −)	1	TN
( +)	( −)	0	FP
( −)	( +)	0	FN

*TP* true positive, *TN* true negative, *FP* false positive, *FN* false negative

The table defines true positive, true negative, false positive, and false negative in AI classification. In cases where both AI and GT are masked, if the DICE coefficient of AI is less than 0.15, the result is considered a false negative. If the DICE coefficient of the AI mask is greater than or equal to 0.15, then the AI masking is considered a true positive

### Model Training and Overview

**Fig. 3:**
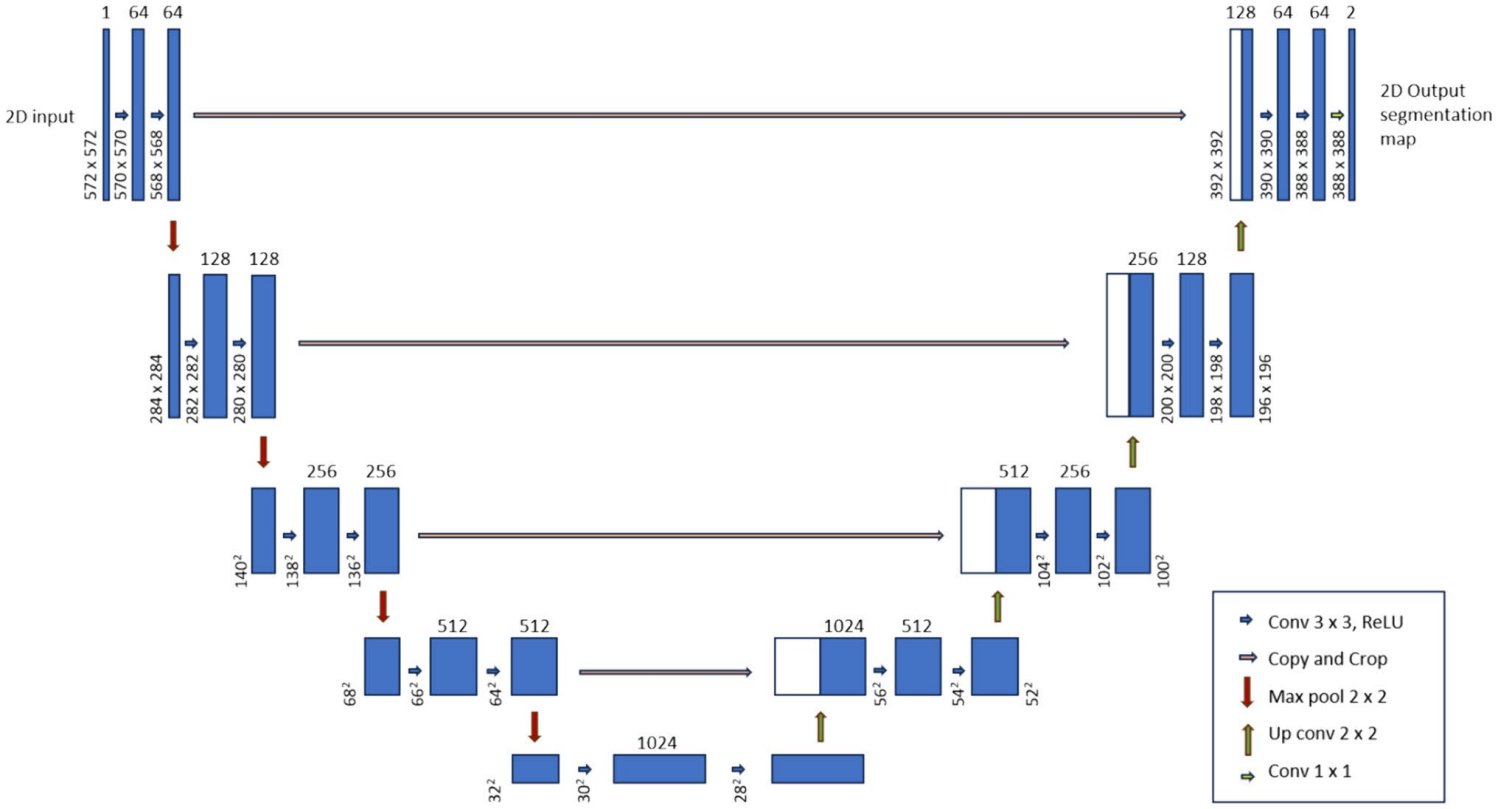
Illustration of the 2D U-Net architecture. The diagram presents the architecture of a 2D U-net, a type of encoder-decoder network. This network features a contracting path on the left, which compresses the height and width of the input images, and an expansive path on the right, which restores the images to their original dimensions. Each blue box represents a multi-channel feature map, while the white boxes indicate the concatenated feature maps copied from the contracting path. The number of channels is indicated above each box, and the dimensions of the image (*x*–*y*-size) are shown along the left edge of each box. The arrows depict various operations detailed in the legend to the right

Our models (model 1 and model 2) utilize a 2D U-Net-based neural network [20] with Efficient-Net b6 serving as the backbone to predict the segmented mask of skull fracture. Our 2D U-Net model architecture is illustrated in Fig. 3. Its design incorporates a contracting path to capture context, juxtaposed with a symmetric expanding path, which facilitates precise localization. In enhancing the network, successive layers replace traditional pooling operations with up-sampling operators, thereby refining the output resolution. For training the model, we used data augmentation methods like flipping and rotating images. The loss function combined Dice loss and focal loss, which is half Dice loss and half focal loss. This combination helps in dealing with unbalanced data and improves the model’s ability to classify difficult examples. We used an adaptive moment estimation (Adam) optimizer with parameter settings of β1 = 0.9 and β2 = 0.999, and a CosineAnnealingLR scheduler with parameter settings of *T*_max = 8 and eta_min = 3e − 6. The model was trained with Nvidia RTX A6000 GPU, with minibatches of size 16, initial learning rate of 5e − 4, and running for 200 epochs, with an approximate duration of 3 days.

### Post-processing Rules

Two post-processing rules were applied to reduce the false-positive rate:

Rule A: Clusters smaller than 78 voxels were excluded.

Rule B: Lesions appearing in less than four continuous slices were excluded.

The threshold of 78 pixels and four continuous slices was determined by examining the entirety of the dataset to establish an approximate cut-point.

### Observer Study—Binary Classification

A two-session observer study was conducted using the test dataset. In session one, each participant was given 671 cases to review. They were required to classify each case as either 1 (fracture) or 0 (no fracture), without AI assistance. In session two, which occurred 1 month after session one, the same 671 cases were reviewed by observers with AI assistance. Timestamps were recorded to measure the diagnostic duration in both sessions.

The participants involved one neuroradiologist (up to 13 years of experience), one neuroradiology fellow, one second-year radiology resident, two neurology fellows, and one emergency physician (up to 6 years of experience).

### Diagnostic Performance and Statistical Analysis

Confusion matrices were calculated using the following equations for binary classifications. For segmentation performance, DICE (Dice coefficient score) was calculated. We employed McNemar’s test to evaluate the differences between two models in terms of their diagnostic performance for skull fractures. To compare the human sensitivity and specificity without AI assistance to that with AI assistance, a two-proportion *z*-test was used.

The diagnostic duration was presented as the mean ± standard deviation. A paired *t*-test was conducted to compare the diagnostic duration of the two sessions, with a significance level of *p* < 0.05 indicating statistical significance. To evaluate whether there is a significant difference in diagnostic duration for skull fracture detection between participants with and without AI assistance, an independent two-sample *t*-test was conducted.

### Ethical Considerations

We have obtained approval from an Institutional Review Board and collected only the data necessary for our research objectives. Prior to analysis, we de-identified the data to ensure privacy. Access to the dataset was strictly controlled and limited to participants involved in the research. Furthermore, all team members engaged in the study received training on the importance of patient privacy.

## Results

### Patient Characteristics

The mean age of the 671 patients was 58.88 years old. There were 366 males and 305 females in the test dataset (Table 3).

**Table 3:** Dataset characteristics

	Model 1	Model 2	Model 1 + B
Dataset	T + V (*n* = 1499)	T + V (*n* = 2829)	Test (*n* = 671)
Mean age	51.63 ± 20.51	54.01 ± 20.51	58.88 ± 20.48
Male %	72.44%	82.83%	54.50%

The patient characteristics of the training and validation dataset for model 1, model 2, and test dataset for Model 1 + B are presented. They are similar in distribution. *T* + *V* training and validation datasets

### Diagnostic Performance of AI Model

Confusion matrices for Model 1 and Model 2 and their combinations with post-processing rules are shown in Table 4. Model 1, which was trained with 1499 fracture-positive cases, achieved a sensitivity (recall) of 0.94, a specificity of 0.87, and a DICE score of 0.65. Model 2, which was trained with additional 1330 false-positive cases from Model 1, showed a sensitivity (recall) of 0.79, a specificity of 0.90, and a DICE score of 0.46. Compared to Model 1, Model 2 had a slightly elevated specificity (*p* = 0.17) but a marked decrease in sensitivity (recall) (*p* < 0.01).

**Table 4:** Confusion metrics for Model 1 and Model 2 and their combinations with post-processing rules

	Sensitivity (recall)	Specificity	DICE	F1 score
Model 1	0.94	0.87	0.65	0.80
Model 1 + A	0.94	0.93	0.65	0.87
Model 1 + B	0.94	0.99	0.63	0.94
Model 1 + AB	0.90	0.99	0.62	0.94
Model 2	0.79	0.90	0.46	0.75
Model 2 + A	0.78	0.96	0.45	0.82
Model 2 + B	0.72	0.99	0.43	0.82
Model 2 + AB	0.69	0.99	0.41	0.81

The best performing combination is Model 1 plus rule B (recall rate of 0.94 and a specificity of 0.99), which achieved the highest F1 score

After applying both post-processing rules on Model 1, the sensitivity (recall) decreased slightly from 0.94 to 0.90 (*p* = 0.32, compared to Model 1), but the specificity increased to 0.99 (*p* < 0.01). DICE score became 0.62, respectively. When we applied only Rule A to Model 1, the sensitivity (recall) and DICE score remained the same (0.94 and 0.65, respectively), but the specificity increased slightly from 0.87 to 0.93 (*p* < 0.01). Applying only Rule B resulted in the best F1 score (0.94), with a sensitivity (recall) of 0.94, a specificity of 0.99 (*p* < 0.01), and a DICE score of 0.63.

Regarding Model 2, applying both post-processing rules led to decreased sensitivity (recall) (0.69, *p* = 0.11, compared to Model 2) and DICE score (0.41), but marked increased specificity (0.99, *p* < 0.01). Applying only Rule A resulted in a minimal decrease in sensitivity (recall) (0.78) and DICE score (0.45), but an increase in specificity (0.78, *p* < 0.01). When only Rule B was applied, the results showed a decreased sensitivity (recall) (0.72, *p* = 0.267) and DICE score (0.43), but an increased specificity (0.99, *p* < 0.01).

### Diagnostic Performance and Duration of Human Readers Without AI Model Assistance

The radiology fellow achieved a sensitivity (recall) of 76.47% and 94.41%, with a diagnostic duration of 20.00 s per case. The radiology second-year resident achieved a sensitivity (recall) of 86.47% and 99.00%, with a diagnostic duration of 21.62 s per case. The neurology fellow A achieved a sensitivity (recall) of 48.82% and 87.62%, with a diagnostic duration of 26.64 s per case. The neurology fellow B achieved a sensitivity (recall) of 75.29% and 98.20%, with a diagnostic duration of 21.50 s per case. The neuroradiologist achieved a sensitivity (recall) of 75.88% and 94.01%, with a diagnostic duration of 10.97 s per case. The emergency physician achieved a sensitivity (recall) of 44.71% and 97.41%, with a diagnostic duration of 12.20 s per case. The AI model showed a sensitivity (recall) of 87.06% and a specificity of 98.60%, respectively. Table 5 presents the diagnostic performance and duration of human readers with and without AI assistance, where the AI model is the combination of Model 1 and Rule B.

**Table 5:** Diagnostic performance of various specialists and the AI model

Model unassisted vs. model assisted (*n* = 671)													
	Radiology fellow		Radiology R2		Neurology fellow A		Neurology fellow B		Neuroradiologist		Emergency physician		AI
	Without AI	With AI	Without AI	With AI	Without AI	With AI	Without AI	With AI	Without AI	With AI	Without AI	With AI	AI alone
Sensitivity	76.47%	**86.47%**	48.54%	**86.47%**	48.82%	**81.40%**	57.06%	**75.29%**	75.88%	**91.18%**	44.71%	**73.53%**	87.06%
Specificity	94.41%	**98.40%**	96.40%	**99.00%**	87.62%	**94.39%**	94.41%	**98.20%**	94.01%	**95.41%**	97.41%	**99.20%**	98.60%
Duration (s/case)	20.00 ± 7.33	**10.50 ± 1.49**	21.62 ± 7.05	**11.25 ± 6.67**	26.64 ± 6.10	**8.81 ± 7.19**	21.50 ± 7.65	**10.00 ± 9.18**	10.97 ± 4.75	**9.75 ± 4.31**	12.20 ± 6.27	**11.48 ± .57**	4.20
*p*	< 0.01*		< 0.01*		< 0.01*		< 0.01*		< 0.01*		< 0.01*		

Sensitivity and specificity of all participants improved and the diagnostic duration (mean ± standard deviation) significantly reduced after the AI’s assistance. The rows on the left of the table represent the unassisted model, while the rows on the right (with bold text) signify the model-assisted scenarios

*indicates a significant difference in diagnostic duration with and without AI assistance

### Diagnostic Performance and Duration of Human Readers with AI Model Assistance

**Fig. 4:**
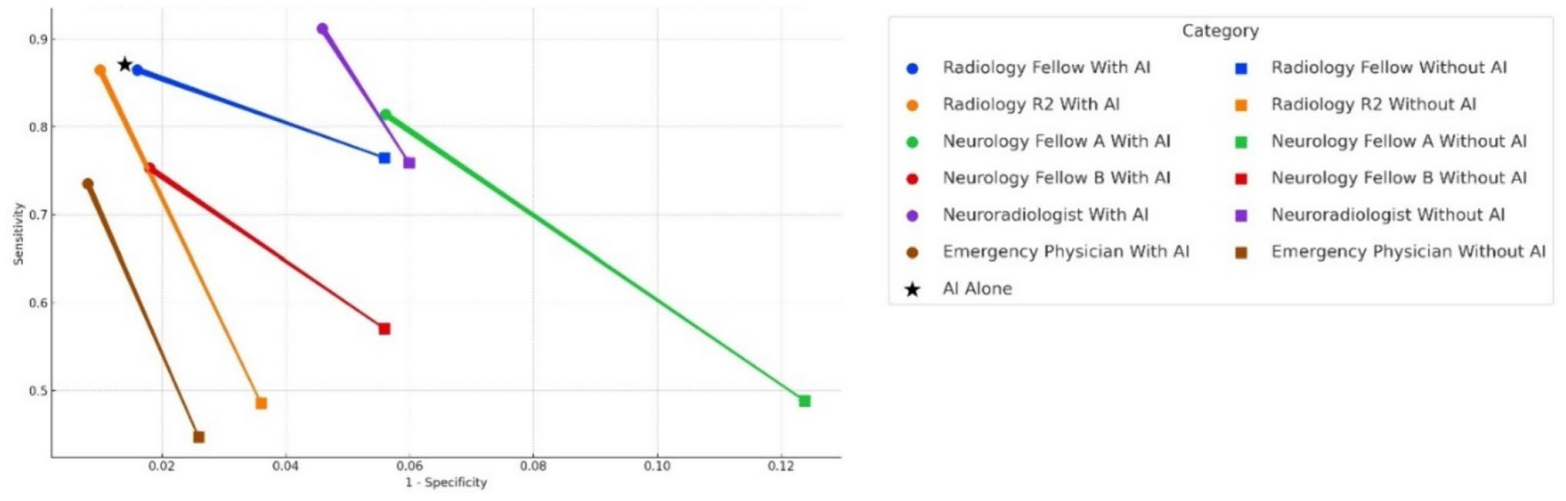
Diagnostic performance of various specialists with and without AI assistance and AI alone. The figure illustrates the diagnostic performance of various medical specialists when aided by artificial intelligence (AI) compared to their performance without such assistance. Each specialist is represented by symbols of the same color, where the square denotes performance without AI support, and the circle represents AI-assisted performance. The lines connecting the two performances within each specialist category facilitate a direct comparison. The asterisk symbol (*) denotes the diagnostic performance enhanced by AI. It is observable from the figure that the inclusion of AI assistance correlates with improved diagnostic performance across all specialist groups. A 1-month washout period was instituted between the two diagnostic assessments

**Fig. 5:**
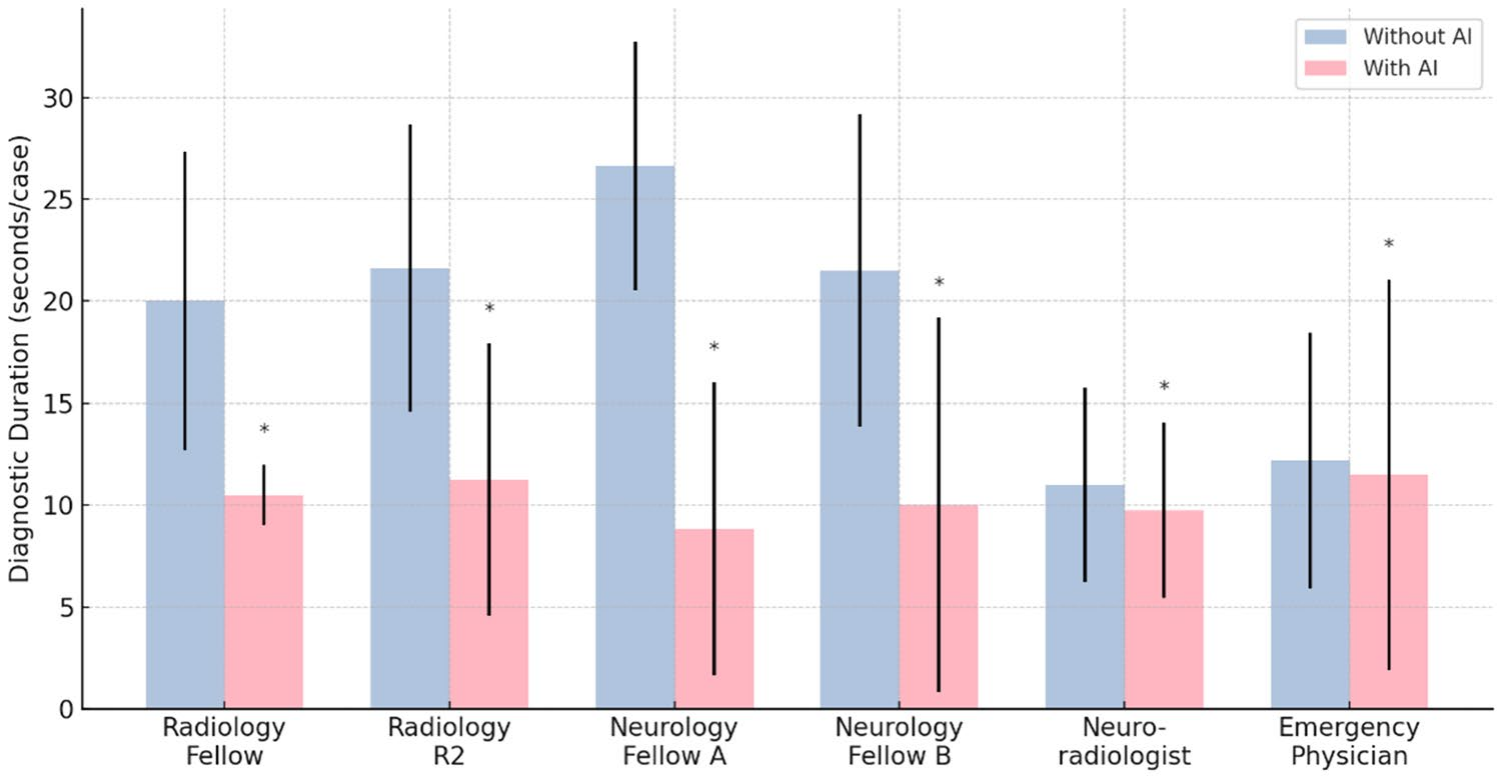
Comparison of diagnostic duration of various specialists with and without AI assistance. The graph depicts the diagnostic duration for skull fractures by various specialists under conditions with and without AI assistance. It is evident that the diagnostic time is significantly reduced when AI support is provided, highlighting the efficacy of AI in aiding the diagnostic process. The pink bars represent the diagnostic duration for skull fractures with AI assistance, while blue bars indicate the duration without AI support. The vertical lines denote the standard deviation. The presence of an asterisk (*) symbolizes statistical significance, with a *p*-value of less than 0.05

With assistance, all participants showed improved performance. The radiology fellow achieved a sensitivity (recall) of 86.47% and 98.40%, with a diagnostic duration of 10.50 s per case. The radiology second-year resident achieved a sensitivity (recall) of 48.54% and 96.40%, with a diagnostic duration of 11.25 s per case. The neurology fellow A achieved a sensitivity (recall) of 81.40% and specificity of 94.39%, with a diagnostic duration of 8.81 s per case. The neurology fellow B achieved a sensitivity (recall) of 57.06% and 94.41%, with a diagnostic duration of 10.00 s per case. The neuroradiologist achieved a sensitivity (recall) of 91.18% and 95.41%, with a diagnostic duration of 9.75 s per case. The emergency physician achieved a sensitivity (recall) of 73.53% and 99.20%, with a diagnostic duration of 11.48 s per case. Significant decreases (*p* < 0.01) in diagnostic duration were noted in all participants after AI assistance. The standard deviation decreased in radiologists but increased in neurologists and the emergency physician. Figures 4 and 5 represent the diagnostic performance and diagnostic duration of human participants with and without AI model assistance.

## Discussion

In this study, we evaluated the diagnostic performance of AI models and human readers in detecting skull fractures on brain CT images. The key distinguishing factors of this study include the following: (1) Both post-operative and pediatric patients were not excluded and with larger pool of cases in the training dataset, facilitating AI training under more realistic conditions; (2) two post-processing rules were employed to reduce false positives; and (3) comparative analysis of diagnostic performances and duration between human readers and AI.

### Diagnostic Performance of AI Model

In this study, two strategies were employed to combat false positives. The first strategy involved training Model 2 using false positives from Model 1. The second strategy utilized two post-processing rules.

As depicted in Table 4, Model 1 demonstrated superior sensitivity (recall) and DICE score compared to Model 2, while Model 2 displayed marginally increased specificity. It is indeed unexpected that Model 2’s performance was less impressive, despite being trained with false positives from Model 1. These cases are more challenging compared to regular negative cases, which could potentially disrupt the AI’s original logic in making judgments, resulting in labeling with less logical reasoning.

For instance, reviewing cases that have undergone surgery can be challenging as they often closely resemble skull fractures, especially in an axial view. At first, the AI might misclassify these as fractures, but through learning, it is taught to discern otherwise, leading to some uncertainty in its judgement. Consequently, the model experienced a decrease in sensitivity (recall) and DICE, yet it saw an enhancement in its specificity.

The model was trained and validated on a diverse dataset, including cases that are typically challenging for radiologists, such as subtle fractures and those obscured by artifacts. The architecture employed is a 2D U-Net, where the input images are flat 512 × 512 pixel slices. Clinically, accurately determining whether an image indeed shows a fracture often necessitates examining several consecutive slices and possibly reconstructing coronal and sagittal views. Although we incorporated adjacent slices above and below the original axial view into a three-channel input, the model fundamentally remains 2D. The 2D approach might still be prone to errors in complex cases.

Therefore, exploring the use of a 3D model in the future could potentially yield better results by allowing for a more comprehensive analysis of the spatial relationships and complexities inherent in skull fractures. However, the adoption of such a model is not without drawbacks. Firstly, 3D models are not typically equipped with pretrained weights, creating a considerable limitation. Secondly, the diminutive nature of fractures raises doubts about the efficacy of a 3D model’s broad-range search. It is plausible that such extensive scanning might not invariably result in improved performance.

### The Impact of Post-processing Rules on Performance

About the post-processing rules, Rule A excluded clusters smaller than 78 voxels, while Rule B excluded lesions appearing in less than four continuous slices. Both rules are designed to reduce false alarms. After applying post-processing rules, both models showed improved specificity, while sacrificing sensitivity (recall) and DICE score. The combination with the highest F1 score was Model 1 plus Rule B, which achieved high sensitivity (recall), specificity, and DICE score.

Integrating Rule A into the models only yielded a slight increase in specificity, indicating that Rule A can reduce false alarms without strongly impacting sensitivity (recall) and DICE score, possibly due to tiny non-fracture lesions. Conversely, adding only Rule B to the models notably boosted specificity, implying Rule B effectively excludes certain non-fracture lesions which are large enough but not long enough such as arachnoid granulations, despite potentially missing some fractures. In certain cases, while the fracture lines are very thin, they indeed span more than four slices. Due to the simultaneous application of Rule A and Rule B, even legitimate fractures could potentially be filtered out. The differing impact of Rule A and B suggests that fracture lesion shape may affect AI performance.

Although the DICE score is not perfect, the primary objective of this AI model is to alert radiologists to potential overlooked fractures. This rationale justifies our use of a segmentation approach for conversion into a binary classification, even with a low DICE threshold. The goal is achieved if the fracture’s location is identified, enabling radiologists to thoroughly examine the relevant areas.

### False Positives (FP) and False Negatives (FN) Incorrectly Identified by AI

**Fig. 6:**
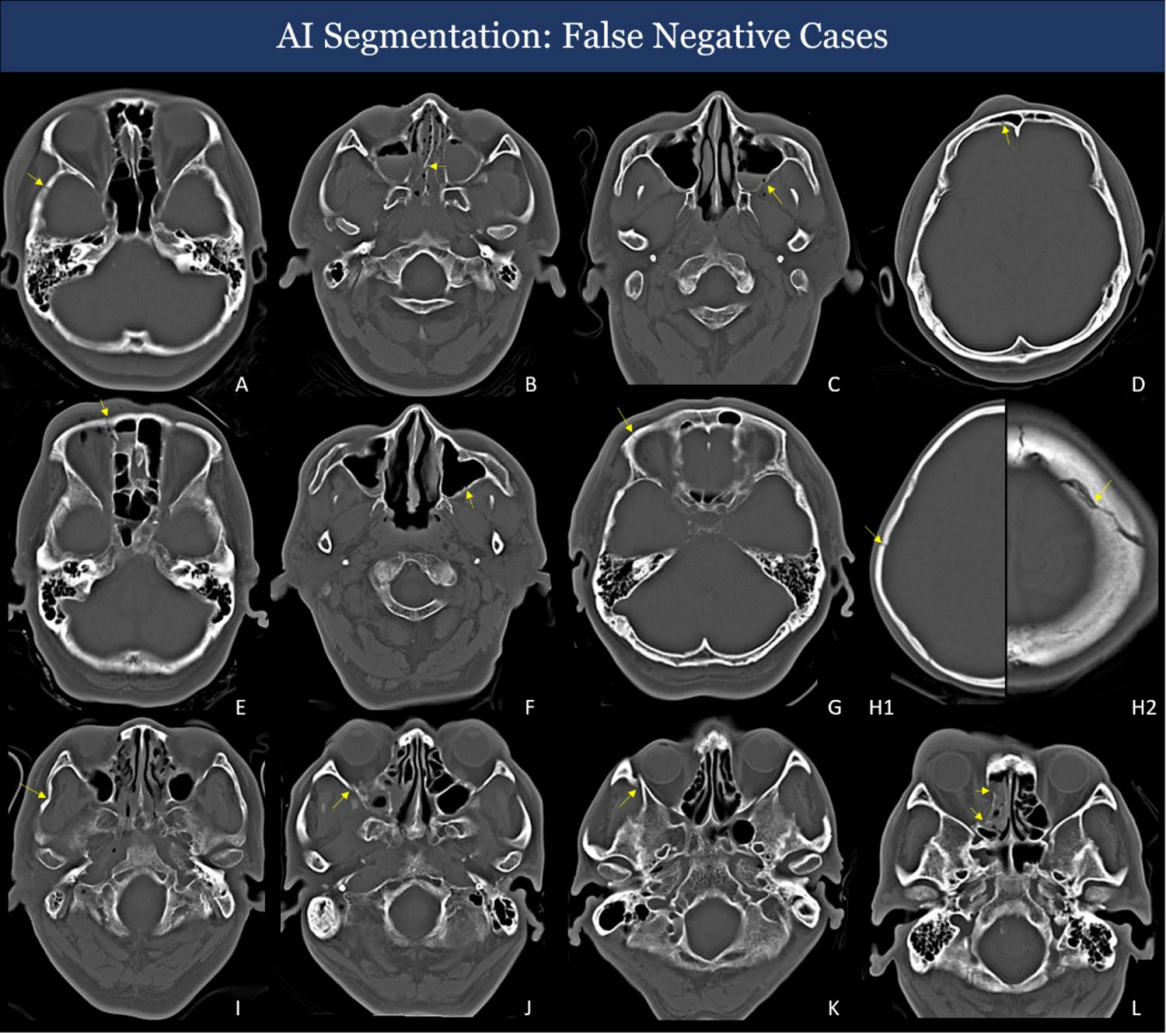
Representative images of false-negative predictions of the model. The figure illustrates numerous instances of false negatives that escaped detection by the AI system. Some have indirect signs, such as swelling of soft tissues, sinus opacification, and asymmetry in the positioning of the orbits. Right temporal bone fracture (**A**). Nasal septum and inner wall of right mastoid sinus fracture (**B**). Lateral wall of the left maxillary sinus (**C**, **F**). Fracture of frontal sinus (**D**). Fracture of ethmoid sinus (**E**, **L**). Superior orbital wall fracture (**G**). Parietal/frontal bone fracture (H1/H2). Right zygomatic arch fracture (**I**). Right lateral orbital wall fracture (**J**, **K**)

Based on Fig. 6, we have noticed that the AI model may generate false-positive cases. Some cases even apparently display indirect signs such as hemosinus, soft tissue swelling, or orbital protuberances. As a result, it is crucial to conduct a thorough assessment of patients with indirect signs and consider their clinical history before reaching a definitive diagnosis.

**Fig. 7:**
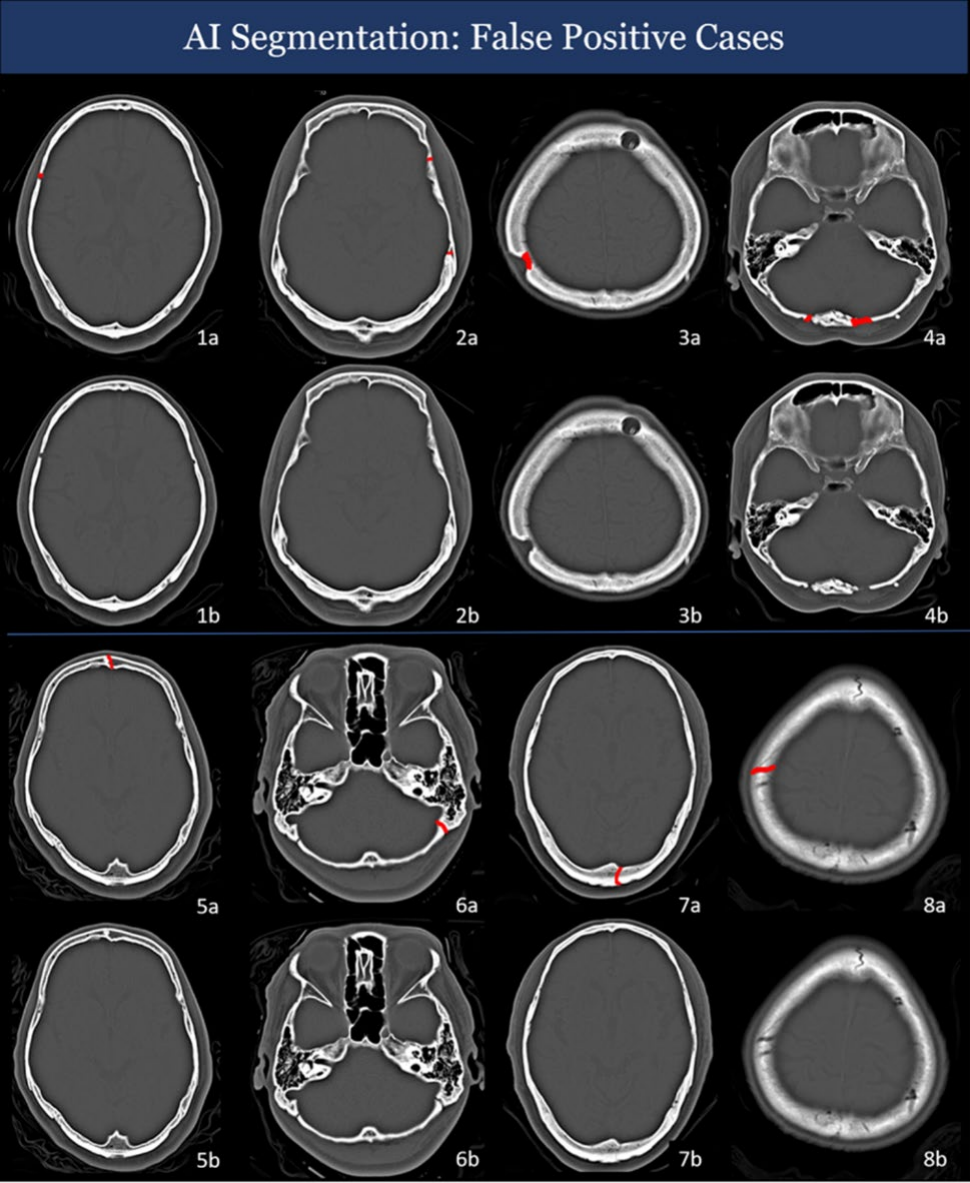
Representative images of false-positive predictions of the model. False-positive cases in AI segmentation. All the red lines in the top row represent the predicted fractures, while the bottom row shows the original images without AI segmentation. Prominent right coronal suture (**1a**, **1b**). Left coronary suture and emissary vein (**2a**, **2b**). Burr holes (**3a**, **3b**). Suboccipital craniectomy (**4a**, **4b**). Frontal suture (**5a**, **5b**). Prominent lambdoid suture (**6a**, **6b**). Parietal emissary vein (**7a**, **7b**) (**8a**, **8b**)

In Fig. 7, there are several cases that AI misclassified them as fracture-positive cases. In summary, most of incorrect segmentation point to sutures, post-operative change, or emissary veins. This finding is consistent with previous studies [21, 22]. Although we cannot understand the methodology of AI prediction in the black box, most of the cases in Fig. 7 lack symmetry. Therefore, we speculate symmetry may be an important clue to the model.

### Observer Study 1—Diagnostic Performance Without AI Assistance

In comparison to human participants, the AI model demonstrated superior performance, boasting the highest sensitivity (recall) (87.06%) and specificity (98.60%), surpassing the best human results. The observers labeled 671 cases in a single period. However, in regular clinical practice, the volume of brain CT scans to be reviewed may be considerably less. Such an intensive review session could potentially lower focus and increase errors, potentially undervaluing human performance.

Compared to AI, human specificity remains within an acceptable range, although the sensitivity (recall) is considerably lower. Radiologists with greater experience significantly surpassed others in terms of sensitivity (recall). The neuroradiologist was the most efficient in interpretation, achieving a comparable specificity to AI in half the time of her counterparts.

### Observer Study 2—Diagnostic Performance with AI Assistance

AI assistance significantly improved the diagnostic performance and reduced diagnostic duration. However, most human performances have not surpassed that of AI. The most impacted is the sensitivity (recall) of less experienced readers, which has nearly doubled. The neuroradiologist still possess the highest sensitivity (recall) among human readers. The specificity of human readers has also increased. Furthermore, the duration of diagnosis has been reduced to half or even one third of the original time.

AI has the capability to expedite the diagnostic process by automatically identifying lesions and visualizing them. By bolstering diagnostic accuracy and significantly reducing the diagnostic time, the integration of AI assistance in the diagnostic process holds great promise for improving patient care [23].

### Comparison with Previous Researches

We have compiled a table (Table 6) to compare various details of studies on skull fractures, including our own study, which is listed in the bottom row.

**Table 6:** Comparison with previous researches

Title	Author	Published time	Modality	Site	Interval	Exclusion criteria	Total case number	Training dataset	Validation dataset
Classification based on the presence of skull fractures on curved maximum intensity skull projections by means of deep learning	Jakob Heimer	2018	Calvarial CT MIP	Skull	2014–2017	Shattered skulls lacking resemblance to physiological anatomy, visible residues from surgical intervention, age below 18 years	75	0.5	0.5
Deep learning algorithms for detection of critical findings in head CT scans: a retrospective study	Sasank Chilamkurthy	2018	Head CT	Head ICH	2011–2017	Post-OP defect; absence of non-contrast (plain) axial series covering complete brain; patients younger than 7 years	Qure25k datasets 21,095, CQ500 dataset 214 + 277		Qure25k dataset + CQ500 dataset
Skull R-CNN: a CNN-based network for the skull fracture detection	Zhuo Kuang	2020	CT	Skull			45	25	10
Identifying fatal head injuries on postmortem computed tomography using convolutional neural network/deep learning: a feasibility study	Jack Garland	2020	PMCT	Head	2018–2020	1. All suspicious, homicidal, and pediatric deaths (age < 10) due to potential legal issues 2. All cases with signs of decomposition 3. All cases with neurosurgical procedures	50	40	
Transfer learning for an automated detection system of fractures in patients with maxillofacial trauma	Maria Amodeo	2021	CT	Skull	2000–2020		208	148 CT	30
Automated identification of skull fractures with deep learning: a comparison between object detection and segmentation approach	Wei Shan	2021	CT	Skull	2016–2020		4782	4168	
A study on 3D deep learning-based automatic diagnosis of nasal fractures	Yu Jin Seol	2022	CT	Skull		> 2 days	2535 (1350 normal/1185 fractured)	864/758	216/190
Automatically diagnosing skull fractures using an object detection method and deep learning algorithm in plain radiography images	Tae Seok Jeong	2022	Plainfilm	Skull	2015–2018	Fracture on plain radiographs could not be found, previous surgery, comminuted fractures	741 patients, 2026 films		
Deep learning-assisted diagnosis of pediatric skull fractures on plain radiographs	Jae Won Choi	2022	Plainfilm	Skull	2013–2019	Previous head surgery	413 + 95	0.7	0.1
Predicting skull fractures via CNN with classification algorithms	Md Moniruzzaman Emon	2022	Head CT	Skull			142 cases	50%	20%
Artificial intelligence application in skull bone fracture with segmentation approach	Our study	2024	CT	Skull	2010–2022		30,000 normal + 1499 fracture	1499, 2200	333, 629

Title	Testing dataset	AI model	Gold standard	Accuracy	AUC	Sensitivity	Specificity	Threshold
Classification based on the presence of skull fractures on curved maximum intensity skull projections by means of deep learning		VIDI	Manual		0.965	91.4	87.5	0.75 (sen100%, spe 72.5%)
Deep learning algorithms for detection of critical findings in head CT scans: a retrospective study		Natural language processing (NLP) algorithm	Manual (8, 12, 20 years)		0.92, 0.96	0.9, 0.81/0/95, 0.87	0.77, 0.90/0.86,0.90	Varying the threshold
Skull R-CNN: a CNN-based network for the skull fracture detection	10	Faster R-CNN	Manual			High		0.5
Identifying fatal head injuries on postmortem computed tomography using convolutional neural network/deep learning: a feasibility study	10	CNN	Autopsy	92.5 (training), 0.7 (testing)				0.1, 0.3, 0.5
Transfer learning for an automated detection system of fractures in patients with maxillofacial trauma	30	ResNet50	manual (2 people)	80%	0.82	0.76	V	0.99
Automated identification of skull fractures with deep learning: a comparison between object detection and segmentation approach	614	YOLOv3, AttentionU-Net	Manual (7 people up to 15 years)	85.96% (YOLOv3), 88.26% (modified AttentionU-Net)		83.33%, 82.80%	80.65%, 88.73%	
A study on 3D deep learning-based automatic diagnosis of nasal fractures	270/237	3D-ResNet:residual neural networks (3D-ResNet34 and ResNet50)	Manual	86.2%, 87.6%	93.4%, 94.5%	86.4%, 87.5%	86.8%, 87.8	
Automatically diagnosing skull fractures using an object detection method and deep learning algorithm in plain radiography images		RetinaNet		V		V	V	0.1
Deep learning-assisted diagnosis of pediatric skull fractures on plain radiographs	0.2	YOLOv3	Cranial CT	0.92 (internal dataset)	0.094, 0.069, 0.008	81.10%	91.30%	0.43
Predicting skull fractures via CNN with classification algorithms	30%	CNN (transfer learning based): nXception, InceptionV3, ResNet50, InceptionResNetV2	Report	F1 0.37, 0.37, 0.39, 0.37	0.55, 0.49, 0.49, 0.50			
		Bagging algorithm (random forest)		F1 0.85, 0.94, 0.90, 0.88	0.85, 0.95, 0.90, 0.87			
		Decision tree (XGBoost)		F1 0.91, 0.96, 0.93, 0.92	0.91, 0.96, 0.93, 0.93			
		Support vector machine (linear SVM)		F1 0.90, 0.93, 0.91, 0.92	0.91, 0.95, 0.93, 0.93			
Artificial intelligence application in skull bone fracture with segmentation approach	671	2D U-Net	3 radiologists	0.97		0.94	0.99	0.15

Our AI model demonstrated robust ability in distinguishing between fracture and non-fracture cases. This is comparable to other studies, yet our model achieves a commendable balance of sensitivity (91%) and specificity (87%), which supports its potential utility in clinical settings.

Additionally, unlike many studies that exclude pediatric patients and those with prior surgeries, our study maintains a natural clinical condition by including these groups. This inclusion enhances the generalizability and applicability of our AI tool across a broader patient spectrum, reflecting real-world scenarios more accurately.

Our dataset is notable not only for its inclusivity but also for its duration and size. Data were collected over a 12-year period, which is relatively long compared to other studies ranging from 2 to 20 years. This extensive collection period allowed us to amass a considerable number of cases, enhancing the reliability of our findings.

Comparatively, most of studies predominantly employ a segmentation approach. This method is favored for its precision in delineating intricate structures such as fractures in skull CT scans. The gold standard for validating AI performance in these studies is mostly radiological CT reports, although one study used autopsy findings (Fig. 2).

### Limitations

Our study faces several limitations that may impact the interpretation and application of its findings.

Firstly, the dataset was confined to a single medical center, which may limit the generalizability of the results across different populations and settings.

Secondly, the composition of the test dataset, specifically the ratio of positive to negative cases, may not accurately reflect real-world clinical scenarios, potentially skewing the AI model’s performance metrics.

Thirdly, the validity of the results could be influenced by a learning effect, as participants evaluated the same dataset twice within a 1-month period, which might have affected their diagnostic accuracy during the second review.

Fourthly, our AI model demonstrated difficulties in distinguishing between actual fractures and artifacts from previous craniectomies. This challenge is exacerbated by the use of 2D images from single slices, which can also pose difficulties for human experts without access to sequential cuts for a more thorough assessment. We suggest that employing a 3D model in future research could potentially mitigate these issues, as it would offer a more comprehensive view of the cranial structure and better differentiate between true fractures and post-surgical changes.

Fifthly, our exploration of different proportions in the training dataset revealed that exclusively training Model 1 with positive cases inadvertently increased the AI’s propensity to predict positives, leading to a higher rate of false positives. To counteract this, we introduced a substantial number of negative cases, referred to as “hard negatives,” into the training process. Although this strategy is based on past successes in enhancing model accuracy and reducing false positives, it did not yield the expected outcomes in this instance. We hypothesize that complications related to post-craniectomy scenarios might have influenced this result. Future studies might benefit from incorporating typical cases, which could help refine the model’s accuracy further by providing a more balanced and realistic training environment.

## Conclusions

In summary, the skull fracture detection model, based on a segmentation approach, has shown promising results in enhancing diagnostic accuracy and efficiency for radiologists and clinicians. However, it is important to acknowledge AI’s limitations and potential risks. For a comprehensive and effective diagnosis, AI-generated results should always be contextualized and cross-referenced with human expertise.

## Supplementary Information

Below is the link to the electronic supplementary material.

Supplementary file1 (DOCX 675 KB)

## Data Availability

The datasets produced or scrutinized throughout the course of this study are accessible from the corresponding author, subject to a reasonable request.

## Abbreviations

AI: Artificial intelligence
CT: Computed tomography
TP: True positives
TN: True negatives
FP: False positives
FN: False negatives
DICE: Dice coefficient
