# Supplementary material for: Artificial Intelligence Application in Skull Bone Fracture with Segmentation Approach
Source: J Imaging Inform Med. 2024 Jul 1;38(1):31–46. doi: 10.1007/s10278-024-01156-0 (PMC11811319; doi:10.1007/s10278-024-01156-0)
Supplement: Supplementary file 1 — Supplementary file1 (DOCX 675 KB) [file 10278_2024_1156_MOESM1_ESM.docx]

**Appendix**

**Appendix 1 schematic diagram (A, B) and examples (1a-3b) of segmentation labeling.**


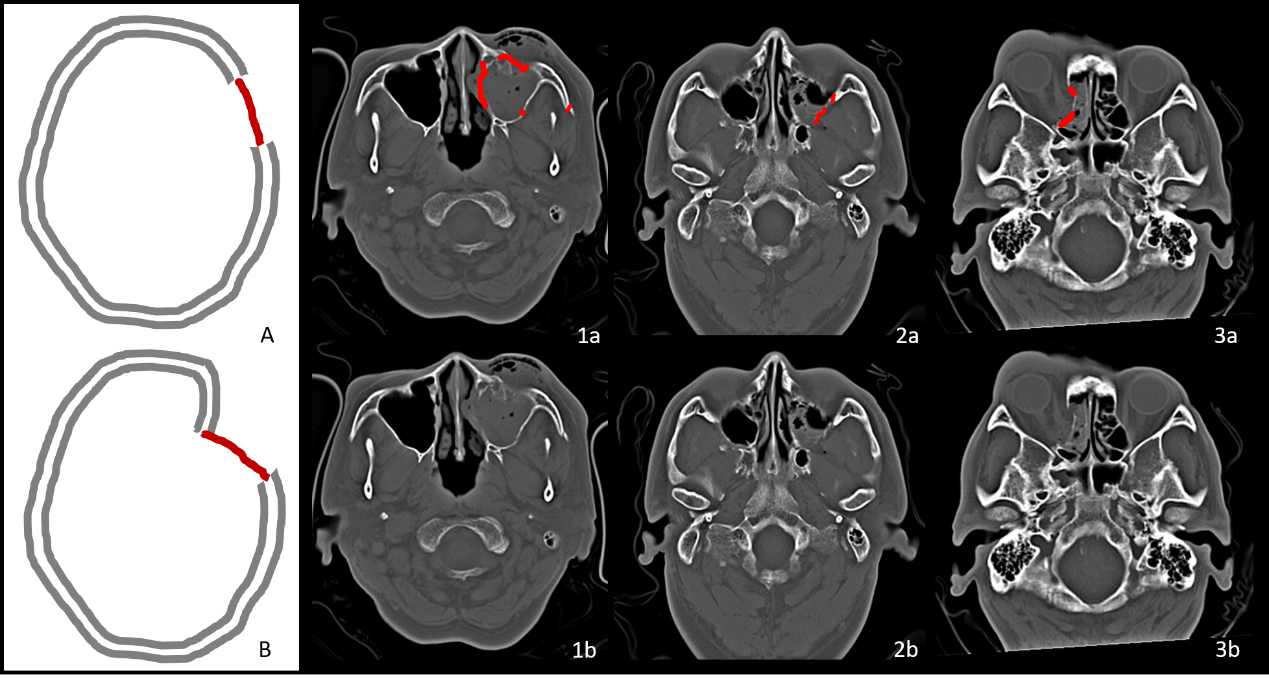


Schematic diagrams (A) and (B) illustrating fracture segmentation in larger cortical defect cases using coordinate annotation of the shortest distance. The red lines in the top row represent the segmentation lines, while the bottom row shows the original images. The fracture segmentation was drawn using a 6-pixel brush size.

**Appendix 2. Pediatric population in our dataset**


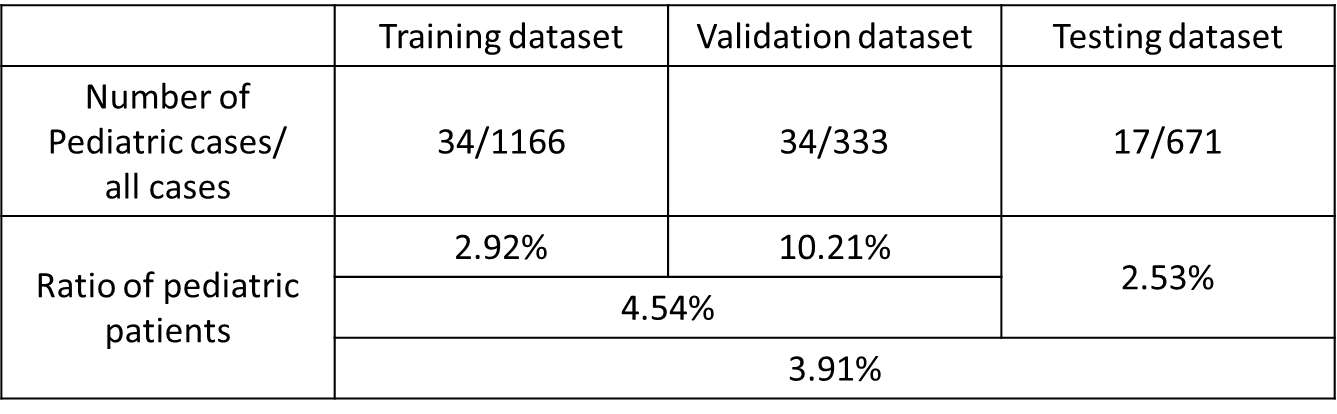


Within our combined training and validation dataset, there are a total of 68 pediatric patients, constituting 4.54% of the dataset (with 34 out of 1166 in the training dataset and 34 out of 333 in the validation dataset). In the testing dataset, which comprises 671 cases, there are 17 pediatric patients, making up 2.53% of this dataset. Overall, there are 85 pediatric patients across all datasets, accounting for 3.91% of the total 2170 cases.

**Appendix 3. The Impact of Post-processing Rules on Performance**

Overall, enhancements to Model 1 and Model 2 generally improved specificity without compromising sensitivity, indicating robust enhancements in model performance, particularly in specificity metrics.

1. Model 1 vs Model 2:

Model 1 displayed significantly higher sensitivity (p<0.01) for detecting skull fractures, while Model 2 showed marginally better specificity (p=0.17) for identifying patients without fractures.

1. Model 1 with post-processing rules:

Model 1+A: Improved specificity significantly over Model 1 (p=0.0058), with identical sensitivity between the two.

Model 1+B and Model 1+AB: Both models showed no significant change in sensitivity compared to Model 1, but both demonstrated significantly better specificity (p<0.01).

1. Model 2 with post-processing rules:

Model 2+A: No significant difference in sensitivity; however, specificity improved significantly (p<0.01).

Model 2+B and Model 2+AB: Sensitivity slightly decreased (not significantly), while specificity significantly improved (p<0.01).

**Appendix 4. Overall human performance with and without AI performance**

Overall Human Performance With and Without AI Assistance:

- Sensitivity without AI: 59.21%
- Sensitivity with AI: 82.39%
- Specificity without AI: 94.04%
- Specificity with AI: 97.44%
- Statistical Significance for Sensitivity and Specificity: p < 0.01, indicating significant improvements in both sensitivity and specificity with AI assistance.

Comparison of Human vs. AI Model Performance (Without AI Assistance):

- AI Model Sensitivity: 90.96%
- AI Model Specificity: 98.82%
- Human Sensitivity: 59.21%
- Human Specificity: 94.04%
- Sensitivity and Specificity p-values: <0.01, demonstrating superior performance of the AI model in detecting both the presence and absence of skull fractures.

Comparison of Human With AI Assistance vs. AI Model:

- Sensitivity p-value: 0.0057, indicating the AI model's higher sensitivity.
- Specificity p-value: 0.0579, showing no significant difference, although closely approaching statistical significance.

Diagnostic Duration Comparisons:

- Without AI Assistance: The t-test shows the AI model significantly reduces the assessment time compared to human diagnosis without AI (t-statistic: 54.84, p-value: <0.01).
- With AI Assistance: Even with AI assistance for humans, the AI model alone shows a more rapid diagnostic process (t-statistic: 28.99, p-value: <0.01), underlining the efficiency of the standalone AI model.
